# The development of a keratoacanthoma following injection of hyaluronic acid–based dermal fillers into the lips

**DOI:** 10.1093/skinhd/vzae022

**Published:** 2025-02-24

**Authors:** Emma Mackender, Erin Kamp, Nicole Hendrix, Nastassia Nardini, Paul Drake

**Affiliations:** Department of Dermatology, University Hospitals Sussex NHS Foundation Trust, Brighton General Hospital, Brighton and Hove, UK; Department of Dermatology, University Hospitals Sussex NHS Foundation Trust, Brighton General Hospital, Brighton and Hove, UK; Department of Dermatology, University Hospitals Sussex NHS Foundation Trust, Brighton General Hospital, Brighton and Hove, UK; Department of Pathology, University Hospitals Sussex NHS Foundation Trust, Brighton and Hove, UK; Department of Dermatology, University Hospitals Sussex NHS Foundation Trust, Brighton General Hospital, Brighton and Hove, UK

## Abstract

This case report illustrates an unusual case of a keratoacanthoma (KA) developing on the lips following repeated injections with hyaluronic acid-based dermal fillers. Hyaluronic acid dermal fillers dominate nonsurgical aesthetic treatments in the UK due to their effectiveness, while also being relatively safe. The vast increase in various filler products has resulted in an increase in adverse events, although these likely remain under-reported. There have been no previously reported cases of KA directly post-hyaluronic acid injections, and KAs are not currently considered a side-effect. The patient in our case had several risk factors for developing a KA, including high sun exposure and smoking, as well as a history of repeated injections performed by a lay practitioner. Moreover, poor injection technique and repeated trauma to the lips from the injections are also likely to be implicated. Given the all-time high increase in demand for cosmetic dermal fillers, we believe that KA should be considered a possible adverse reaction of dermal fillers, particularly in patients with other significant risk factors for KA development.

## Teaser

We present an unusual finding of a keratoacanthoma (KA) developing on the lips following injection of hyaluronic acid–based dermal fillers. Dermal fillers dominate aesthetic non-surgical treatments in the UK. The vast increase in various filler products has resulted in an increase in adverse events, although these likely remain underreported. The patient in our case had several risk factors for developing KA, including trauma from repeated injections. Given the all-time high increase in demand for cosmetic dermal fillers, we believe that KA should be considered a possible adverse reaction, particularly in patients with other significant risk factors for KA development.

**What is already known about this topic?**

- Keratoacanthomas (KAs) are common benign neoplasms with many risk factors.
- Recently, we have seen a vast increase in the ­number of dermal filler products available and an associated high volume of reported of adverse reactions.
- A recent systematic review found that foreign body granulomas are the most common lesion on histopathological analysis.
- Currently, there are no other reported KAs that have developed directly as a result of hyaluronic acid-based dermal fillers.

**What does this study add?**

- Our case is an unusual finding of a KA developing on the lips following injection of hyaluronic acid-based dermal fillers.
- This finding is significant as KAs are not currently considered a possible adverse reaction.
- Given the all-time high demand for cosmetic dermal fillers, we believe that KA should be considered a possible adverse reaction, particularly in patients with other significant risk factors for KA development.

##  

Keratoacanthoma (KA) is a common, benign neoplasm ­arising from the hair follicle.^1^ KAs present as rapidly ­enlarging, well-demarcated keratotic nodules with a crateriform appearance.^1^ Although most KAs regress spontaneously, they are clinically indistinguishable from squamous cell carcinomas (SCCs) and are therefore treated surgically, with an excellent prognosis.^1^ KA usually occurs in individuals with a lighter skin phototype aged >60 years; it is twice as common in men than in women. The main risk factors are considered to be previous trauma to the site, sun exposure, immunosuppression, viruses and carcinogens.^1^ We present an unusual case of a KA developing on the lower lip following injection of ­cosmetic hyaluronic acid dermal filler products.

**Figure 1:**
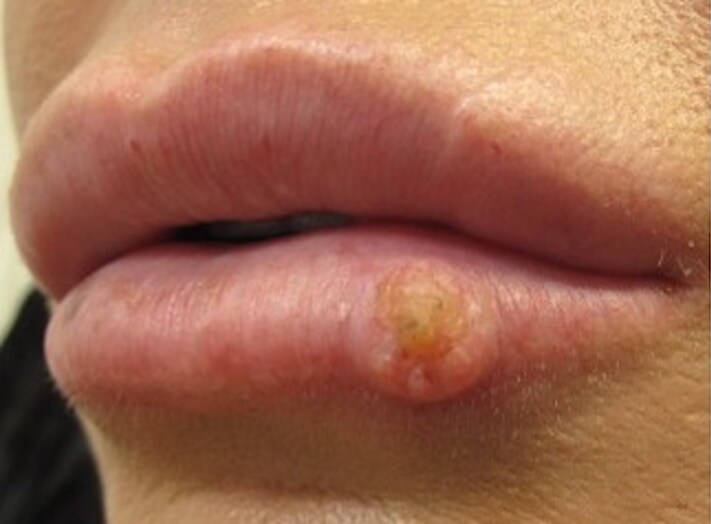
Keratoacanthoma on the left lower lip.

A woman in her thirties was referred to our dermatology department from general practice, with a 6-week history of a rapid enlargement of a longstanding small lesion (Figure 1). At the time of presentation, the patient had a long history of repetitive hyaluronic acid dermal filler injections to the lips. The brand of filler used, volume and frequency of injections was unknown, but the injections were given by a lay practitioner. The last injection was a few months prior to presentation. The patient also had a history of COVID-19 vaccination in the weeks before presentation and had recent dental veneers. The patient was otherwise well, with a past medical history of fibromyalgia. However, she had a history of high sun exposure with high sunbed use (>100 sessions) and living abroad. She also smoked and vaped. On examination, there was a 6 mm × 7 mm painful, firm, well-­demarcated keratotic nodule on the left lower lip. The differential diagnoses based on history and exam included SCC, granulomatous reaction to lip filler, infection (including atypical infection) and KA.

**Figure 2:**
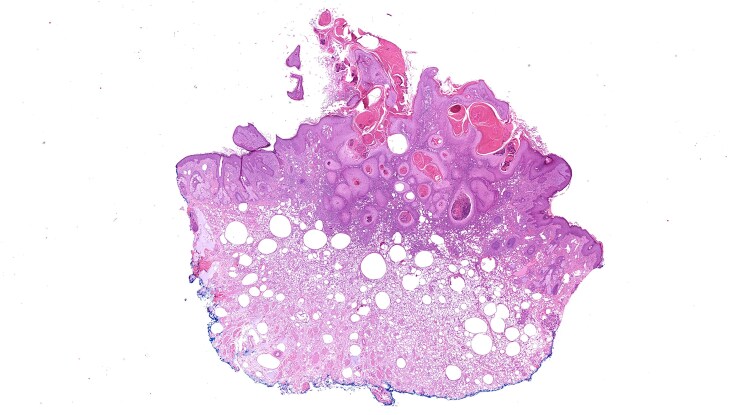
Locally arising squamoproliferative lesion composed of lobules of keratinocytes with glassy cytoplasm and mild cytological atypia, extending to the upper dermis consistent with keratoacanthoma. In the background there are scattered vacuoles, a dense histiocytic infiltrate and filler deposition, in keeping with hyaluronic acid. Haematoxylin and eosin; original magnification × 2

**Figure 3:**
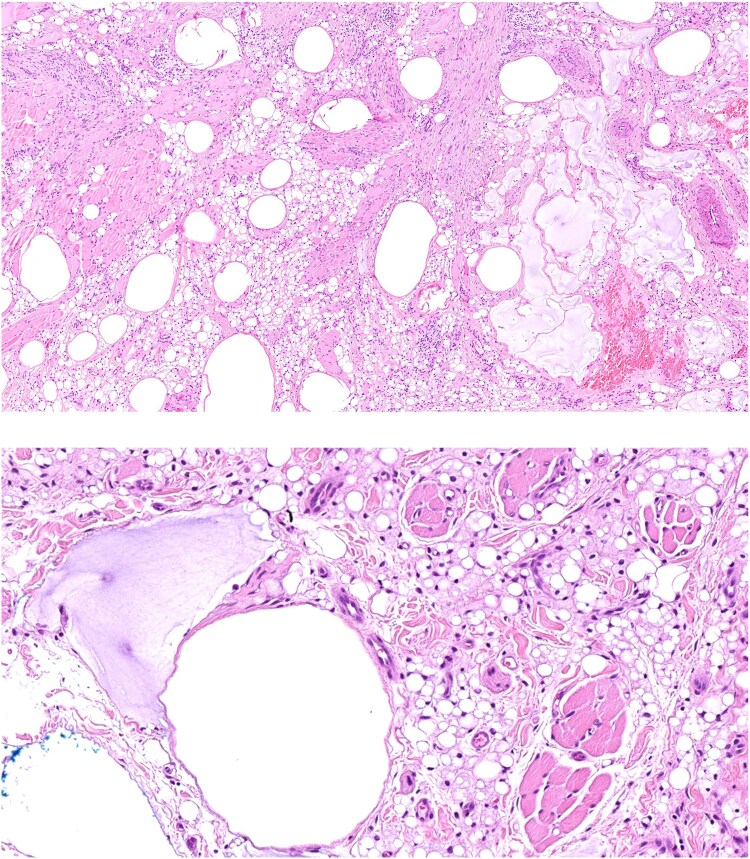
Amorphous basophilic material in keeping with hyaluronic acid associated with a dense histiocytic infiltrate comprising foamy histiocytes and pseudolipoblasts, features consistent with a reaction to cutaneous filler. Haematoxylin and eosin, original magnification x 10 (top) and × 20 (bottom).

The lesion was biopsied on initial presentation and the patient was brought back for a further appointment to fully surgically excise the lesion. Histopathology was consistent with KA (Figures 2, 3). Of note, there were also histopathological features consistent with a reaction to cutaneous lip filler. Thus, histopathology concluded that this was a KA plus a reaction to cutaneous filler. The lesion was completely excised with clear margins and the patient was discharged.

Hyaluronic acid-based dermal fillers dominate ­nonsurgical aesthetic treatments in the UK, mostly due to their effectiveness and reversibility, and also their ‘favourable safety profile’.^2^ In recent years we have seen a vast increase in the number of dermal filler products available and a significant increase in patient demand, described as being at ‘an all-time high’.^3^ Thus, there has been increased reporting of adverse reactions caused by various filler products. Early adverse events may consist of erythema, bruising, pain and oedema, while delayed adverse events may consist of nodule or foreign-body granuloma formation, and migration of filler material.^2^ KAs are not currently considered an adverse event.

A recent systematic review found that, on histopathological analysis, foreign-body granulomas are the most common lesion found on the lips caused by hyaluronic acid injections.^3^ Although the incidence is considered rare, it is not uncommon for patients to present with ‘unknown or incomplete medical and cosmetic treatment history’.^2,4^ Patients themselves may not link new lesions with previously injected temporary filler, and therefore may not seek medical help. Thus, it is likely that delayed adverse events are under-­reported, which may include KA. Furthermore, the patient in our case also offered limited information regarding their injections.

Currently, no KAs that have developed directly as a result of hyaluronic acid dermal filler injections have been reported. However, there has been an isolated report of a KA-like reaction following a single hyaluronic acid dermal filler injection to the forehead.^4^ This lesion was described histologically as a ‘keratoacanthoma-like lesion’ likely due to the presence of nonatypical keratinocytes. This contrasts with our histology, which clearly described a typical KA lesion (Figure 2). There have also been two reports of KA secondary to collagen-based filler.^3^ Treatment was successful with surgical excision, although one case report described the use of acitretin, but this failed to completely remove the lesion.^3^

In the UK there is a lack of regulation of all dermal fillers and there is no legal requirement to be trained to administer the injections.^5^ Poor injection technique has also been implicated in the development of foreign-body granulomas.^6^ Thus, injection technique may have also been implicated in the development of the KA in our patient. Furthermore, the trauma to the lips from the repeated injections is likely to also have contributed.

The patient in our case had several risk factors for developing KA, including high sun exposure and smoking. However, given the histological findings of KA, as well as filler reaction, we believe that her history of hyaluronic acid injections in combination with her other risk factors were the major provoking factors for the development of the KA.

To conclude, given the worldwide increase in use of dermal fillers, KA should be considered a possible sequela of repetitive dermal filler injections, particularly in patients with other significant risk factors for KA development. Excision and histological analysis of the lesion is essential.

## Data Availability

No new data were generated or analysed in support of this research.
